# The conjunctival fungal microflora of horses in a North Queensland tropical environment and their in vitro susceptibilities to antifungal agents

**DOI:** 10.1007/s11259-023-10119-9

**Published:** 2023-04-06

**Authors:** Phu Mo Mo, Jacqueline Picard, Bruce Gummow

**Affiliations:** 1 College of Public Health, Medical and Veterinary Sciences, James Cook University, Queensland, Australia; 2 Faculty of Veterinary Science, University of Pretoria, Pretoria, South Africa

**Keywords:** Horse, Fungi, Tropical, Commensal microflora, Keratomycosis, Risk factors, Conjunctiva

## Abstract

Fungi are ubiquitous in the environment and part of the commensal microflora on the conjunctiva of equine eyes. North Queensland, being tropical, presents an ideal environment for fungi growth. When the cornea is injured, fungi can invade the corneal stroma, resulting in keratomycosis. The objectives of this study were to determine the fungal species specific to the eyes of horses in the Townsville region; to investigate the potential risk factors associated with the presence of fungi; and to test their susceptibility to antifungals to create an empirical guide for treatment. The eyes of forty ophthalmologically normal horses from James Cook University were sampled throughout the summer months of December 2017, January 2018, and January and February 2020. Cultured fungi were identified morphologically, and their identity confirmed by comparing partial 18sRNA DNA sequences with the NCBI nucleotide database. Minimum inhibitory concentration testing of common antifungal medications was performed. Sixty-one out of eighty conjunctival samples grew fungi, and 21 different fungi genera were isolated. The most common genera were *Aspergillus* (18%, 26/141), *Curvularia* (14%,20/141), *Rhodotorula* (12%,17/141) and *Penicillium* (12%,17/141). No significant association was found between age or environmental factors and fungal culture status. Most fungi were highly susceptible to voriconazole and ketoconazole but resistant to fluconazole and amphotericin B. This adds to the body of evidence on which species of fungi are present as normal ocular microflora of horses living in tropical regions of Australia, and an avenue for treating them.

## Introduction

Corneal disease in horses is very common and poses a significant threat to vision retention, which can jeopardize their future use as performance animals (Miller et al. 2010). Horses are predisposed to ocular trauma and ulceration due to the prominent conformation of the ocular globe and deficiencies of the innate immuno-protective barriers in the tear film (Andrew et al. 1998). Because of deficiencies in the innate immune protective barriers of the tear film horses may not develop an adequate response against fungi present in the conjunctiva and the cornea. Other contributing factors may be a lack of integrity and stability of the precorneal tear film, a large corneal surface, and low resting temperature of the cornea (Galán et al. 2009; Galera & Brooks 2012; Cafarchia et al. 2013; Khosravi et al. 2014).

Fungi can be found as a conjunctival microflora of the eye and the prevalence is especially high in horses compared to other domestic species due to exposure to hay, straw, and bedding harbouring fungi (Andrew et al. 2003; Samuelson et al. 1984). Moreover, the concentration of fungi in the environment is very high in tropical regions which have a hot and humid environment, like in tropical North Queensland (Thew and Franzco 2008).

The ubiquitous presence of fungi on the surface of the conjunctiva and cornea combined with suspected defects of the equine corneal immune system in mounting a protective immune response against fungi, make the infection of the horse cornea more likely than in other domestic animal species (Samuelson et al. 1984; Andrew et al. 1998; Brooks 1999).

Horses have a higher prevalence of ocular infection than other species, apart from humans (Gilger 2016). Among equine ophthalmologic cases, keratomycosis is a common disease, accounting for up to 2–10% of cases (Andrew et al. 1998; Reed et al. 2013).

Despite these facts, no study has been done on equine ocular fungal microflora and keratomycosis in tropical North Queensland (Townsville). Knowledge of the conjunctival microbiota is important for the treatment of keratomycosis because causal organisms are believed to be opportunistic pathogens resident in the conjunctival pouch. However, there are few reports documenting the equine ocular fungal microbiota. A study by Rosa et al. (2003) documented fungal organisms’ resident in the eyes of horses in Rio de Janeiro, Brazil, a location with similar climatic and environmental conditions to Townsville. We hypothesised that in-vitro anti-fungal susceptibility patterns are associated with fungal species and regions where they are found and would therefore be different in tropical north Queensland compared to those reported elsewhere in Australia and internationally. The objectives of this study were to determine the species of fungi specific to the eyes of horses in the Townsville region; to investigate the potential risk factors associated with the presence of fungi; and to test their susceptibility to common antifungals in order to create an empirical guide for treatment. An additional objective was to compare these results to results obtained from previous clinical cases of keratomycosis in Townsville from 2013 to 2017.

## Materials and Methods

### Study population

This study was performed ethically, and the approval was granted by James Cook University Human and Animal Ethics Committees (approval No. A2670) to carry out a study on the teaching horse population of James Cook University (JCU), Townsville campus, Queensland. The population studied were drawn from the 80 horses permanently housed and managed at JCU, which were regarded as representative of horses in this part of Northern Queensland. This population of horses was further chosen because of its availability and proximity to the laboratories used in the study.

A cross-sectional observational study was carried out using convenience sampling based on availability of horses that were not currently being used for teaching purposes. Twenty horses were sampled in December 2017 and again in January 2018, and 20 different horses were sampled in January 2020 and again in February 2020. Hence the study comprised 40 horses that were each sampled twice (a month apart) over two time periods. This provided 80 eye samples that could be analysed, which was the primary sampling unit for the study. Horses were kept on the JCU Townsville campus during December 2017, January 2018 and January 2020. However, the horses were moved to another location in Townsville (Kelso) in February 2020 for management reasons. Hence, the 20 eyes sampled in February 2020 were at another location to those of the previous 60 eyes sampled.

No horses were kept in stables, which is considered as one of the most important factors that escalate fungi incidence and influence the species of fungi reported in many studies (Moore et al. 1988; Rosa et al. 2003; Whitley and Moore 1984). Most of the horses housed at JCU were adult mares.

A second population used in the study were the existing data on equine keratomycosis cases diagnosed in a single referral equine hospital in Townsville, North Queensland from 2013–2017. This was utilized to compare the fungi associated with these keratomycosis cases and those from equine normal conjunctival microflora in this study.

### Sample collection and storage

Sampling was performed to coincide with the peak incidence of keratomycosis in horses in the southern hemisphere tropics, which according to Townsville Vet Clinic (TVC) records, was from January to March in the monsoonal months. For sample collection, horses were placed in normal standing physical restraint stocks or stanchion stalls. All horses were clinically healthy at the time of sample taking. The eyes were examined by an equine veterinarian to ensure they were free from any lesions. All the samples were taken by an equine veterinarian, without topical anaesthesia or nerve block, from the conjunctival fornix. A commercial bacteriological swab soaked in normal saline was used and rolled along the conjunctival fornix, avoiding contact with vibrissae, eyelids, or eyelashes. This procedure was carried out on a single eye of each horse using a new swab for each horse. Except for two horses, one that had a lesion in the left eye and another where the swab was contaminated, the left eye was always sampled.

Ocular examination was again performed on every horse 24 h after the taking of samples to ensure there had been no adverse effects from the sampling process.

All swabs were labelled and coded with a unique number for identification. Immediately after collection, swabs were transported in Amies transport medium on ice packs (4 °C) to the BSL-2 laboratory in the Discipline of Veterinary Science, James Cook University. The age of the horse, climatic condition, and season were recorded.

### Laboratory assays

#### Culture and morphological identification

The same method was used to culture and identify fungi from swabs collected from the affected cornea in clinically affected horses as well as from conjunctival swabs. Each swab was rolled in a zig-zag pattern across the surface of Sabouraud’s dextrose agar with 0.5% chloramphenicol (Cell Biosciences, Australia) and 0.1% kanamycin (Merck, Australia). The plates were incubated at 30ºC under aerobic conditions for 7 days and examined daily for evidence of growth. To purify the colonies, the hyphal tip of each colony type was collected, and stab inoculated into potato dextrose agar (PDA) (Cell Biosciences, Australia). It was incubated at 30 °C and examined for growth 24 and 48 h later. Colonies were sub-cultured on PDA at least twice to get a pure culture to be used for DNA extraction and sequencing. The colonies were examined under a stereomicroscope (Olympus SZX10) for aerial fruiting bodies and, if present, cellulose adhesive tape impressions were made from the surface of a fungal colony and stained with lactophenol cotton blue dye (ProLab™ Diagnostics, Australia). Isolates with no fruiting bodies were treated in two ways: either by incubating in the light or by slide cultures in tap water agar, consisting of 1.5% agar (Oxoid Products, UK) and tap water. The fungi were identified by their macro- and micro-morphological characteristics of fruiting structures, according to Kidd et al. (2016). and with the aid of available websites such as Mycology Online (https://mycology.adelaide.edu.au/) and Doctor Fungus (http://www.mycosesstudygroup.org/). These websites are extensively used for morphological diagnosis as they provide images and information relating to fungi of clinical importance. Fungi isolates were stored in microtubes with beads and storage broth (MicrobankTM, Prolab Diagnostics UK) at -80 °C until sequencing could be carried out.

#### Antifungal susceptibility testing

Minimum Inhibitory Concentration (MIC) was determined for common antifungal agents on each fungal isolate. The antifungals itraconazole (Sigma-Aldrich), voriconazole (Sigma-Aldrich), ketoconazole (Sigma-Aldrich), amphotericin B (Sigma-Aldrich), nystatin (Nilstat, Aspen, Australia) and fluconazole (Diflucan, Pfizer) were used for this purpose. The MIC testing process was carried out according to the standard procedure used at the Clinical-&-Laboratory-Standards-Institute (2017). Brain heart infusion broth (CellBiosciences) is used as the nutrient source for this test. The obtained data were recorded, and breakpoints of MIC 50 and MIC 90 were calculated per fungus genus and compared to published data using the data published by the University of Adelaide and others (Clinical-&-Laboratory-Standards-Institute 2017 + ; Pearce et al. 2009). The same data set was used to calculate microbiological cut-offs for what constituted resistant and susceptible fungi.

#### Identification of fungal isolates by partial sequencing and analysis of fungal specific amplicons

A realtime polymerase chain reaction (qPCR) was performed on a micPCR (Biomolecular Systems) using DNA extracted (QuickGene DNA tissue kit S, Kurabo) from isolates that had been purified three times by culture. Like the DNA extraction, the qPCR was carried out using the manufacturer’s instructions with intercalating SYBR dye (Sensifast™ Hi-Rox kit, Bioline) and two sets of universal fungi primers that amplify the interspacer regions between the small subunit and large subunit ribosomal genes. Namely, forward primer 28SR6R (5’-AAG-TAT-AAG-TCG-TAA-CAA-GG-3’) and reverse primer LR1 (5’-GGT-TGG-TTT-CTT-TTC-CT-3’) and forward primer ITS1 (5’-TCC-GTA-GGT-GAA-CCT-GCG-G-3’) and reverse primer ITS4 (5’-TCC-TCC-GCT-TAT-TGA-TAT-GC-3’) (Meyer et al. 2019). The acquired DNA was sent to the Australian Genome Research Facility (AGRF) at Gehrmann Laboratories, Research Road, University of Queensland, Brisbane, Queensland, Australia for Sanger sequencing. AGRF offers different services, and their “unpurified” PCR Product (PD +) service was chosen for analysis. The obtained sequenced results were examined, cleaned up, and aligned using sequence alignment tools, and then compared using the nucleotide blast to the fungal database on the NCBI website (https://www.ncbi.nlm.nih.gov/genomes/FUNGI/funtab.html).

## Data analysis

Descriptive statistics were used to compare the variation in fungi between the two different timeframes. Categorical variables are reported as frequencies. The association between fungi culture status (negative or positive) and age, geography, temperature, and rainfall was assessed using the Fisher’s exact test. Horses were classified into two age groups (≤ 15 years and > 15 years). Microsoft Office Excel (version 2004) and the database program (Epi Info version 7.2.3.1) were used for data analysis. The confidence level was set at 95% and statistical significance was set at p < 0.05.

## Results

### Demographic factors

The sample population of the healthy horses were 38 mares and 2 geldings of which 37 were Thoroughbreds and 3 Standardbreds. No ponies or draft horses were present in the study. As ninety-five percent of the population studied were female no conclusions can be drawn concerning gender and the prevalence of fungi. The small number of Standardbreds in the study meant no conclusions could be drawn regarding an association with breed.

The age of the horses ranged from 7 years old to over 30 years of age. The majority of the horses (89%, 25/28) were under 20 years old. The age range with the highest frequency was 13 to 15 years (36%, 10/28), while the number of horses older than 20 years was only 3 (11%, 3/28). No association could be found between horses > 15 years and the isolation of fungi from their eyes (p ≤ 0.05).

Examination of the existing data on equine keratomycosis cases diagnosed in a single referral equine hospital in Townsville from 2013–2017 found 29 horses had been diagnosed by clinicians with keratitis. From these 29 horses, 14 horses had had fungi isolated (Table 1). The ages of these 14 horses suffering from fungal keratitis ranged in age from 6 to 20 years with an average age of 12 years which is like the age range for horses in the current study. Hence, when comparing the two cohorts of horses, age was unlikely to be a confounder.

**Table 1:** Different genera or species of fungi obtained from conjunctival fornix specimens of single eyes from Townsville horses and fungi species isolated from keratomycosis cases (2013–1017) in Townsville

Species	Occurrence Frequency (2018)		Occurrence Frequency (2020)		Fungi isolated from keratomycosis cases (2013–1017)	
	Number	Percentage	Number	Percentage	Number	Percentage
*Aspergillus*	16	21.62	10	14.92	1	7.14
Curvularia*	13	17.57	7	10.45	2	14.29
*Fusarium*	12	16.22	3	4.48	1	7.14
*Rhodotorula*^§^	7	9.46	10	14.93	0	0
*Penicillium*	8	10.81	9	13.43	0	0
Exserohilum rostratum*	3	4.05	8	11.94	3	21.43
Cladosporium*	7	9.46	5	7.46	1	7.14
*Nigrospora*	0	0	5	7.46	0	0
Alternaria alternata*	1	1.35	1	1.49	2	14.29
*Paecilomyces*	1	1.35	0	0	0	0
*Phoma*	0	0	4	5.97	0	0
Epicoccum*	2	2.7	0	0	0	0
*Trichophyton*	0	0	2	2.99	1	7.14
*Trichoderma*^§^	1	1.35	0	0	0	0
*Cystobasidium sloofiae*^§^	0	0	1	1.49	0	0
Aureobasidium*	1	1.35	0	0	0	0
*Verticillium*	1	1.35	0	0	0	0
*Rhizopus*	1	1.35	0	0	0	0
*Kodomaea ohmeri*^§^	0	0	1	1.49	0	0
*Pseudocercospora kadsurae*^§^	0	0	1	1.49	0	0
*Candida* spp. ^§^	0	0	0	0	1	7.14
Unidentified	0	0	0	0	2	14.29
Total	74		67		14	

^*^ Indicates the black pigmented species; ^§^ Indicates yeasts

### Geographic and environmental factors

In this study, horses were kept at times at two different Townsville locations (JCU and Kelso). A Fisher’s exact test could not show any difference in frequency of ocular fungi between the two different locations within the 2020 cohort of horses. However, a higher frequency of black fungi was isolated from those residing in JCU than Kelso, suggesting that the dematiaceous fungi were more prevalent in the JCU campus environment than Kelso area. This highlights the role that environment may play in seeding the conjunctiva with fungi.

There were no extremes of temperature during either sampling period. The temperature ranged from 22–33 °C in December 2017 and January 2018 and 24.1–33.7 °C in January and February 2020. Rainfall was different between the two sampling periods: December 2017 = 3.4 mm and January 2018 = 96.8 mm and January 2020 = 178.4 mm and February 2020 = 298.8 mm. But no difference could be shown between the frequency of ocular fungi in 2018 and 2020 (p ≤ 0.05).

The 2013 to 2017 hospital records found culture positive keratomycosis cases occurred throughout the year with the lowest numbers in August through to November and the highest numbers in December through to March (46%, n = 6). The dry, cooler months of May through to July accounted for 38% (n = 5) of the cases. However, the number of samples is too low to draw any conclusions from this.

### Frequency of different fungal species

A total of sixty-one out of eighty eye samples had positive fungi culture results, showing the presence of 20 different fungal genera or species (Table 1). Fungi were isolated 141 times, with multiple species being isolated from an eye. One hundred and twenty fungal isolates (85%) were identified as moulds and twenty-one as yeasts (15%). Among the eighty eyes, nineteen eyes gave no growth, (24%), and eight (1310%) eyes produced a single fungal species, while the remaining 53 (66%) eyes had more than one fungal species (87%). In the 2018 cohort, the most frequent genus isolated was *Aspergillus* (22%), then *Curvularia* (18%), followed by *Fusarium (*16%) while in the 2020 cohort *Aspergillus* and *Rhodotorula* ranked equally (15%) followed by *Penicillium* (13%). Twenty different genera were identified in both 2018 and 2020.

In comparison, 14 of the 29 retrospective keratitis cases submitted for culture between 2013–2017 (5 years) to James Cook University had a positive fungal culture and 8 different species were identified with two isolates not being identified (Table 2). The same species obtained from keratomycosis cases were also cultured from normal healthy eyes of horses in our study except for the two yeasts, *Trichosporon* spp. and *Candida* spp. (Table 1). However, the number of black pigmented fungi were proportionally higher in the clinical samples (57%) compared with the conjunctival isolates (27%).

**Table 2:** Number and percentages of fungal genera isolated from the normal eyes of Townsville horses that were susceptible to a specific antimicrobial agent in 2018 and 2020

Fungal spp.	No	KET	ITR	AmB	NYS	VOR	FLU
*Aspergillus*	21	5 (23.8%)	7(38.9%)	1(4.8%)	10(47.6%)	15(88.2%)	2(9.5%)
*Fusarium*	14	5(35.71%)	1(33.33%)	0	5(35.71%)	6(42.86%)	0
*Penicillium*	13	3(23.08%)	2(22.22%)	0	3(23.08%)	8(61.54%)	0
*Exserohilum rostratum*	5	3(60%)	0	1(20%)	4(80%)	4(80%)	2(40%)
*Cladosporium*	10	3(30%)	1(16.67%)	1(10%)	1(10%)	4(40%)	2(20%)
*Curvularia*	17	10(58.82)	3(30%)	3(17.65%)	5(29.41%)	13(76.47%)	1(5.88%)
*Nigrospora*	3	0	0	0	2(66.67%)	0	0
*Phoma*	3	0	0	0	2(66.67%)	0	0
*Alternaria*	1	1(100%)	0	0	0	1(100%)	0
*Epicoccum*	2	0	1(50%)	0	1(50%)	0	1(50%)
*Trichophyton*	2	0	0	0	1(50%)	0	0
*Verticillium*	1	0	1(100%)	1(100%)	0	1(100%)	1(100%)
*Rhizopus*	1	1(100%)	0	0	0	0	0
Yeasts	14	4(28.57%)	1(12.5%)	1(7.14%)	7(50%)	5(35.71%)	0

^*^ No., number of strains isolated belonging to the fungal species; FLU, fluconazole; ITR, itraconazole, KET, ketoconazole; AmB, Amphotericin B; VOR, voriconazole; NYS, Nystatin

### Minimum Inhibitory Concentration

Testing of fungal isolates with these antifungals demonstrated a pattern of high-level resistances in the current study. The fungi were most susceptible to voriconazole, then ketoconazole, nystatin and itraconazole and finally amphotericin B.

The number of moulds and yeasts isolated from the healthy eyes and percentages that are susceptible to different antimicrobial agents are shown in Table 2.

Table 3 provides the minimum inhibitory concentration (MIC) range, (MIC) 50 and 90 values, as well as the percentage of resistant fungal isolates from six different fungal genera which has distinct number of isolates. The cut-off values were determined using previously published data (CLSI, 2017; Pearce et al. 2009; Marangon et al. 2004).

**Table 3:** Minimum inhibition frequency charts for fungal genera with nine or more isolates irrespecitive of species

MIC frequency chart and values for *Aspergillus* species																
Antifungal	total	Number of isolates in each MIC (µg/mL)											MIC_Range_	MIC_50_	MIC_90_	% Resistant
		<	0.12	0.25	0.5	1	2	4	8	16	32	≥ 64				
Ketoconazole	21	3^*^		1^*^	0^*^	1^*^	**1**^**□**^	**2**^**□**^	**4**^**□**^	**1**^**□**^	**2**^**□**^	**6**^**□**^	< 0.3—≥ 64	8	≥ 64	76.19
Voriconazole	21	8^*^	1^*^	2^*^	1^*^	3^*^	**3**^**□**^	**1**^**□**^	**0**^**□**^	**0**^**□**^	**0**^**□**^	**2**^**□**^	≤ 0.06- ≥ 64	0.25	4	28.57
Itraconazole	18	3^*^		2^*^	0^*^	2^*^	**2**^**□**^	**2**^**□**^	**1**^**□**^	**2**^**□**^	**3**^**□**^	**1**^**□**^	≤ 0.06–32	4	32	61.11
Fluconazole	21	1^*^			0^*^	0^*^	1^*^	0^*^	0^*^	0^**□**^	0^**□**^	**19**^**□**^	≤ 0.06- ≥ 64	≥ 64	≥ 64	90.48
Amphotericin B	21	0^*^		1^*^	0^*^	0^*^	**1**^**□**^	**1**^**□**^	**0**^**□**^	**4**^**□**^	**4**^**□**^	**10**^**□**^	≤ 0.125- > 128	32	≥ 64	95.24
Nystatin	21	4^*^		1^*^	2^*^	3^*^	**1**^**□**^	**0**^**□**^	**3**^**□**^	**0**^**□**^	**2**^**□**^	**5**^**□**^	0.125- ≥ 64	1	≥ 64	52.38
MIC frequency chart and values for *Curvularia* species																
Antifungal	total	Number of isolates in each MIC (µg/mL)											MIC_Range_	MIC_50_	MIC_90_	% Resistant
		<	0.12	0.25	0.5	1	2	4	8	16	32	≥ 64				
Ketoconazole	17	9^*^		0^*^	0^*^	1^*^	**1**^**□**^	**0**^**□**^	**2**^**□**^	**0**^**□**^	**1**^**□**^	**3**^**□**^	≤ 0.5—≥ 64	≤ 0.5	≥ 64	41.18
Voriconazole	17	5^*^	2^*^	2^*^	2^*^	2^*^	**0**^**□**^	**1**^**□**^	**0**^**□**^	**0**^**□**^	**0**^**□**^	**3**^**□**^	≤ 0.06- ≥ 64	0.25	≥ 64	23.53
Itraconazole	10	1^*^			2^*^	0^*^	**1**^**□**^	**2**^**□**^	**0**^**□**^	**0**^**□**^	**4**^**□**^	**0**^**□**^	≤ 0.06–32	4	32	70
Fluconazole	17	1^*^			0^*^	0^*^	0^*^	0^*^	1^*^	**3**^**□**^	**2**^**□**^	**10**^**□**^	≤ 0.06- ≥ 64	≥ 64	≥ 64	88.24
Amphotericin B	17	2^*^		0^*^	0^*^	1^*^	**1**^**□**^	**1**^**□**^	**1**^**□**^	**1**^**□**^	**2**^**□**^	**8**^**□**^	≤ 0.125- > 128	32	≥ 64	82.35
Nystatin	17	4^*^		0^*^	1^*^	0^*^	**0**^**□**^	**0**^**□**^	**1**^**□**^	**1**^**□**^	**2**^**□**^	**8**^**□**^	0.125- ≥ 64	16	≥ 64	70.6
MIC frequency chart and values for *Fusarium* species																
Antifungal	total	Number of isolates in each MIC (µg/mL)											MIC_Range_	MIC_50_	MIC_90_	% Resistant
		<	0.12	0.25	0.5	1	2	4	8	16	32	≥ 64				
Ketoconazole	14	4^*^		0^*^	0^*^	1^*^	**0**^**□**^	**1**^**□**^	**3**^**□**^	**1**^**□**^	**2**^**□**^	**2**^**□**^	≤ 0.3—≥ 64	8	32	64.29
Voriconazole	14	1^*^	2^*^	1^*^	2^*^	0^*^	**2**^**□**^	**1**^**□**^	**0**^**□**^	**2**^**□**^	**1**^**□**^	**2**^**□**^	≤ 0.06- ≥ 64	2	32	57.14
Itraconazole	3	0^*^		0^*^	0^*^	1^*^	**0**^**□**^	**0**^**□**^	**0**^**□**^	**0**^**□**^	**0**^**□**^	**2**^**□**^	1- ≥ 64	1	≥ 64	66.67
Fluconazole	14				0^*^	0^*^	0^*^	0^*^	0^*^	**1**^**□**^	**2**^**□**^	**11**^**□**^	16- ≥ 64	≥ 64	≥ 64	100
Amphotericin B	14	0^*^		0^*^	0^*^	0^*^	**1**^**□**^	**1**^**□**^	**0**^**□**^	**0**^**□**^	**1**^**□**^	**11**^**□**^	2- ≥ 64	≥ 64	≥ 64	100
Nystatin	14	3^*^		1^*^	0^*^	1^*^	**0**^**□□**^	**1**^**□**^	**0**^**□**^	**0**^**□**^	**0**^**□**^	**8**^**□**^	≤ 0.3—≥ 64	≥ 64	≥ 64	64.29
MIC frequency chart and values for *Penicillium* species																
Antifungal	tootal	Number of isolates in each MIC (µg/mL)											MIC_Range_	MIC_50_	MIC_90_	% Resistant
		<	0.12	0.25	0.5	1	2	4	8	16	32	≥ 64				
Ketoconazole	13	2^*^		0^*^	0^*^	1^*^	**1**^**□**^	**2**^**□**^	**2**^**□**^	**1**^**□**^	**0**^**□**^	**4**^**□**^	≤ 0.3—≥ 64	4	≥ 64	76.92
Voriconazole	13	3^*^	0^*^	2^*^	1^*^	2^*^	**0**^**□**^	**0**^**□**^	**0**^**□**^	**0**^**□**^	**0**^**□**^	**5**^**□**^	≤ 0.06- ≥ 64	0.5	≥ 64	38.46
Itraconazole	9	1^*^		0^*^	0^*^	1^*^	**0**^**□**^	**0**^**□**^	**0**^**□**^	**2**^**□**^	**2**^**□**^	**3**^**□**^	≤ 0.06- ≥ 64	16	≥ 64	77.78
Fluconazole	13	0^*^			0^*^	0^*^	0^*^	0^*^	0^*^	**1**^**□**^	**0**^**□**^	**12**^**□**^	16- ≥ 64	≥ 64	≥ 64	100
Amphotericin B	13	0^*^		0^*^	0^*^	0^*^	**0**^**□**^	**0**^**□**^	**2**^**□**^	**2**^**□**^	**2**^**□**^	**7**^**□**^	8- ≥ 64	32	≥ 64	100
Nystatin	13	1^*^		1^*^	0^*^	1^*^	**1**^**□**^	**1**^**□**^	**1**^**□**^	**0**^**□**^	**0**^**□**^	**7**^**□**^	0.25- ≥ 64	8	≥ 64	76.92
MIC frequency chart and values for *Cladosporium* species																
Antifungal	total	Number of isolates in each MIC (µg/mL)											MIC_Range_	MIC_50_	MIC_90_	% Resistant
		<	0.12	0.25	0.5	1	2	4	8	16	32	≥ 64				
Ketoconazole	10	2^*^		0^*^	0^*^	1^*^	**2**^**□**^	**1**^**□**^	**1**^**□**^	**0**^**□**^	**0**^**□**^	**3**^**□**^	≤ 0.3—≥ 64	2	≥ 64	70
Voriconazole	10	1^*^	1^*^	0^*^	1^*^	1^*^	**1**^**□**^	**0**^**□**^	**1**^**□**^	**1**^**□**^	**0**^**□**^	**3**^**□**^	≤ 0.06- ≥ 64	2	≥ 64	60
Itraconazole	6	0^*^		0^*^	0^*^	1^*^	**0**^**□**^	**1**^**□**^	**1**^**□**^	**0**^**□**^	**3**^**□**^	**0**^**□**^	≤ 0.06–32	8	32	83.33
Fluconazole	10	1^*^			0^*^	0^*^	1^*^	0^*^	0^*^	**0**^**□**^	**0**^**□**^	**8**^**□**^	≤ 0.06- ≥ 64	≥ 64	≥ 64	80
Amphotericin B	10	1^*^		0^*^	0^*^	0^*^	**0**^**□**^	**1**^**□**^	**0**^**□**^	**2**^**□**^	**0**^**□**^	**6**^**□**^	0.25- ≥ 64	≥ 64	≥ 64	90
Nystatin	10	1^*^		0^*^	0^*^	0^*^	**2**^**□**^	**1**^**□**^	**2**^**□**^	**0**^**□**^	**0**^**□**^	**4**^**□**^	≤ 0.3—≥ 64	8	≥ 64	90
MIC frequency chart and values for *Rhodotorula* species																
Antifungal	total	Number of isolates in each MIC (µg/mL)											MIC_Range_	MIC_50_	MIC_90_	% Resistant
		<	0.12	0.25	0.5	1	2	4	8	16	32	≥ 64				
Ketoconazole	9	2^*^		0^*^	0^*^	1^*^	**0**^□^	**1**^**□**^	**1**^**□**^	**1**^**□**^	**0**^**□**^	**3**^**□**^	≤ 0.3—≥ 64	4	≥ 64	66.67
Voriconazole	9	0^*^	0^*^	0^*^	3^*^	1^*^	**1□**	**0**^**□**^	**2**^**□**^	**0**^**□**^	**0**^**□**^	**2**^**□**^	0.5- ≥ 64	1	≥ 64	55.56
Itraconazole	3	0^*^		0^*^	0^*^	0^*^	**0**^**□**^	**0**^**□**^	**1**^**□**^	**0**^**□**^	**2**^**□**^	**0**^**□**^	0.5–32	8	32	100
Fluconazole	9	0^*^			0^*^	0^*^	0^*^	0^*^	0^*^	**0**^**□**^	**1**^**□**^	**8**^**□**^	32- ≥ 64	≥ 64	≥ 64	100
Amphotericin B	9	0^*^		0^*^	1^*^	0^*^	**0**^**□**^	**0**^**□**^	**1**^**□**^	**1**^**□**^	**2**^**□**^	**4**^**□**^	0.5- ≥ 64	32	≥ 64	88.89
Nystatin	9	2^*^		0^*^	0^*^	1^*^	**0**^**□**^	**2**^**□**^	**1**^**□**^	**2**^**□**^	**0**^**□**^	**1**^**□**^	≤ 0.3—≥ 64	4	16	66.67

^*^ Indicates susceptible isolates ^**□**^ Indicates resistant isolates (in bold)

Tables 4 and 5 show the range of MICs (minimum inhibitory concentrations) for all yeast and fungal isolates along with the values of MIC 50 and 90. The overall resistance percentages for all yeast and fungal isolates are also provided in the tables to help with treatment decisions for keratomycosis in the Townsville region.

**Table 4:** Minimum Inhibitory concentration (MIC) of all fungal isolates

Antifungal name	%S	%R	MIC Range	MIC 50	MIC 90
Ketoconazole	36.11	63.89	≤ 0.3—≥ 64	4	≥ 64
Voriconazole	54.63	45.37	≤ 0.06- ≥ 64	1	≥ 64
Itraconazole	26.67	73.33	≤ 0.06- ≥ 64	4	≥ 64
Fluconazole	11.11	88.89	≤ 0.06 ≥ 64	≥ 64	≥ 64
Amphotericin B	6.48	93.52	≤ 0.12—≥ 64	32	≥ 64
Nystatin	37.04	62.96	≤ 0.3—≥ 64	4	≥ 64

^*^S indicates susceptibility, R Indicates resistant

**Table 5:** Minimum Inhibitory concentration (MIC) of all fungal isolates

Antifungal name	%S	%R	MIC Range	MIC 50	MIC 90
Ketoconazole	28.57	71.43	≤ 0.3—≥ 64	8	≥ 64
Voriconazole	35.72	64.28	0.25- ≥ 64	8	≥ 64
Itraconazole	12.5	87.50	1- ≥ 64	16	32
Fluconazole	-	100	32 ≥ 64	≥ 64	≥ 64
Amphotericin B	7.14	92.86	0.5—≥ 64	32	≥ 64
Nystatin	50	50	≤ 0.3—≥ 64	1	32

^*^S indicates susceptibility, R Indicates resistant

The susceptibility data of different fungi from retrospective keratomycosis cases (2013–2017) to antimicrobial agents are shown in Table 6.

**Table 6:** Number and percentage of fungal genera isolated from keratomycosis cases in Townsville (2013–2017) that were susceptible to a specific antimicrobial agent

Fungal spp.	KET	ITR	AmB	NYS	FLU
*Fusarium*	1(100%)	0	0	0	0
*Exserohilum*	3(100%)	1(100%)	3(100%)	2(66.67%)	2(66.67%)
*Cladosporium*	1(100%)	0	1(100%)	0	0
*Trichosporon*	1(100%)	0	0	1(100%)	1(100%)
*Alternaria*	1(50%)	2(100%)	1(50%)	1(100%)	2(100%)
*Candida*	1(100%)	0	0	0	1(100%)

^*^ FLU, fluconazole; ITR, itraconazole, KET, ketoconazole; AmB, Amphotericin B; NYS, Nystatin

## Discussion

*Aspergillus* and *Fusarium* are reported as the most common causes of keratomycosis (Barnett et al. 2004; Brooks 1999; Stoppini et al. 2003; Lassaline et al. 2010) and were common in the conjunctival swabs from healthy horses in this study (Table 1). However, it was the black pigmented fungal genera, namely *Exoserohilum*, *Alternaria*, *Curvularia* and *Cladosporium* that accounted for the majority (57%) of fungi identified in cases of keratomycosis in Townsville from 2013 to 2017 (Table 2). As shown in Table 1, the prevalence of yeasts was low (15%) with *Rhodotorula* being the most common genus isolated (12%). *Rhodotorula* is often associated with water contamination or poor bedding quality (Khosravi et al. 2014) and despite its presence in the conjunctiva, it does not appear to be an important cause of equine keratomycosis. There was only one isolate each of the yeasts, *Kodomaea ohmeri*, *Pseudocercospora kadsurae, Aureobasidium,* and *Cystobasidium sloofiae*. Their clinical significance is uncertain as only *Candida* and *Trichosporon* species have been implicated in keratomycosis in Townsville. *Fusarium* and *Exserohilum rostratum* have slightly different numbers of isolates but this difference was shown not to be statistically significant.

Since most of the mould genera recovered from normal eyes in this study are similar to those from equine keratomycosis cases, it is likely that fungi in the conjunctival fornix are able to invade the injured cornea (Reed et al. 2013). Although not shown in the Tables, repeated sampling of the same eye yielded different fungal genera in most horses, reinforcing the opinion that fungi in the conjunctival fornix are temporary residents that originate from the environment (Reed et al. 2013).

As ninety-five percent of the population studied were females, no conclusions could be drawn concerning gender. No previous studies mention any differences in the conjunctival flora when comparing the gender of the horses (Hampson et al. 2019). Gender differences have been found in domestic rabbits and pigs (Cooper et al. 2001; Davidson et al. 1994). Also, no significant differences were found between breeds in most studies (Hampson et al. 2019) but one study from Iran found that Caspian miniature horses had high colony forming units (CFU) of fungi per sample compared to other breeds (Khosravi et al. 2014). They believe that this might be due to the horses’ smaller stature, which brings their head and conjunctiva into close contact with feed allowing the saprophytic fungi to be transferred to eyes.

Statistical analysis between two different age groups was done in this study using age groups (< 15 years, > 15 years,) and no association was found between age and frequency of ocular fungal organisms. This statement agrees with many studies (Barsotti et al. 2006; Johns et al. 2011; Hampson et al. 2019; Sgorbini et al. 2008), in which the authors stated that age had no effect on the frequency or type of fungal organisms found. But one study by Andrew et al., (2003), found that there was an increased number of Gram-negative bacterial and fungal isolates in younger animals. This difference may be because of ocular surface defence mechanism differences between age groups (Cooper et al. 2001).

The average rainfall (mm) datasets were very different between the two periods studied, 50 mm, and 239 mm respectively. Even so, no association between the frequency or type of fungi and rainfall was shown in our study, which agrees with other studies (Samuelson et al. 1984; Hampson et al. 2019).

In other studies, seasonal factors played a large role in the prevalence of keratomycosis with the greatest prevalence of disease being in the late summer, early autumn (Hendrix et al. 1995; Gaarder et al. 1998; Grahn et al. 1993). Summer was chosen as the sampling period as this was the time when most of the cases of equine keratomycosis are diagnosed and when environmental conditions are optimal for fungal growth in the environment. It was also at this time when the fungal burden in the pasture or hay is expected to be high. The horses in this study were sampled in December and January, just prior to the expected peak in keratomycosis cases (Gaarder et al. 1998; Grahn et al. 1993). However, 38% (n = 5) of the cases of equine keratomycosis in Townsville (2013–2017) occurred in winter when it is dry, with average daytime temperatures of 26 °C, indicating that cases could be non-seasonal. Laboratory cases however, represented only a small portion of the total cases diagnosed clinically as keratomycosis. They tended to be the more severe and recurrent cases where horse owners more readily agreed to laboratory testing. Thus, the laboratory results are biased and may not reflect the true seasonality of keratomycosis. The cases in dry winter months could be explained by more woody plants being present in the pasture as well as feeding with hay at that time. The woody plants and hay stems can traumatise the cornea allowing fungi to attach to and penetrate the cornea (Brooks 1999). Studies would have to be carried out in the winter months to determine whether there is a seasonal difference in fungal diversity and burden in the conjunctival fornix of horses.

The interpretation of susceptibility to antifungals is difficult since the clinical breakpoints for antifungals are lacking. So, the data from previous publications and CLSI documents were used to interpret the pattern in this study (Clinical-&-Laboratory-Standards-Institute 2017; Pearce et al. 2009; Marangon et al. 2004). In earlier studies, it was reported that fungi isolated from horses’ eyes were generally resistant to fluconazole and ketoconazole and susceptible to natamycin, nystatin, and miconazole (Moore et al. 1988). Other studies reported that most fungi were susceptible to itraconazole and voriconazole, and resistant to fluconazole (Brooks et al. 1998; Ledbetter et al. 2007; Pearce et al. 2009). The latter studies are similar to our findings, where 109 out of 121 fungal isolates were resistant to fluconazole. Antifungal testing of fungi isolated from the ulcerative corneas of keratomycosis cases in Townsville from 2013–2019 also indicated that fluconazole is the least effective antifungal drug (Table 6). Moreover, most studies have shown that equine-origin fungal isolates have poor susceptibility to fluconazole (Brooks et al. 1998; Grahn et al. 1993; Pearce et al. 2009). We found that amphotericin B was not a very effective in vitro agent for the treatment of fungi associated with equine keratomycosis in this study (Table. 6). In this study, fungal isolates displayed high susceptibility to voriconazole and ketoconazole while voriconazole was the most effective drug against moulds in vitro. This agrees with what others have reported in Australia and internationally (Hampson et al. 2019; Pearce et al. 2009).

Voriconazole and itraconazole appear to be a better choice for *Aspergillus* than ketoconazole and fluconazole based on the in vitro susceptibility data generated by this study (Table 3). This agrees with a study from Florida, where *Aspergillus* spp. showed poor susceptibility to fluconazole compared to itraconazole and miconazole (Brooks et al. 1998; Ledbetter et al. 2007). In a recent study done on a human invasive fungal infection, voriconazole appeared to be highly effective as a treatment for human aspergillosis (Warnock 2007). Moreover, Meletiadis et al. (2007) showed that voriconazole has a broader spectrum of activity than other triazole antifungals and evidence of a concentration-dependent sigmoid pattern of fungicidal effect on *Aspergillus* species. Voriconazole can effectively penetrate the cornea in clinically normal horse eyes (Clode et al. 2006). Given orally it will reach 50% of its original concentration in the aqueous humour.

For *Fusarium* spp., natamycin is highly recommended by many studies (Brooks et al. 1998; Ledbetter et al. 2007). But natamycin was not tested in this study due to the lack of availability of the drug. Natamycin is a polyene compound that would be expected to have a similar susceptibility behaviour to amphotericin B and nystatin. Our study demonstrated that, nystatin worked well on *Fusarium*, but amphotericin B did not. Based on the in vitro susceptibility data generated by this study, voriconazole, ketoconazole, and nystatin are the recommended antifungals for *Fusarium* (Table 3).

For *Penicillium* spp., ketoconazole and voriconazole seemed to be better choices than fluconazole and amphotericin B based on the in vitro susceptibility data generated by this study (Table 3). A similar pattern was established in *Exserohilum, Cladosporium,* and *Curvularia* species. The current study demonstrated poorer susceptibility of *Nigrospora* and *Trichophyton* spp. to every antifungal tested. But this might be because only very few isolates of these species were tested. The *Phoma* species of fungi exhibited susceptibility to nystatin only. However, *Phoma* was not isolated in Townsville from the keratomycosis cases.

Only one isolate each of *Epicoccum, Alternaria, Verticillium,* and *Rhizopus* species were tested. All of them, except *Epicoccum*, were susceptible to ketoconazole while itraconazole appeared to work well on *Epicoccum* and *Verticillium.* Since the number tested was very small, the susceptibility pattern of these species cannot be determined.

The yeasts were susceptible to voriconazole and nystatin in this study but less susceptible to fluconazole and Amphotericin B (Table. 5). This study provided evidence that certain costly antifungal medications such as fluconazole and amphotericin B may no longer be effective against most fungi and should be considered as the less ideal drug of choice in treatment plans for equine keratomycosis.

Although ketoconazole proved to be effective in this study, some studies disagree (Chopin et al. 1997; Ledbetter et al. 2007). Ketoconazole is poorly absorbed orally in horses, unless it is administered daily via stomach tube with hydrochloric acid. Furthermore, it inhibits hepatic P450 enzymes reducing enzymic breakdown of other drugs.

These differences in susceptibility patterns between studies might be due to geographic, temporal, or climatic variation and evolving mechanisms of fungal resistance. Fungal communities are adaptive and may develop resistance after being exposed to antifungals, making infections harder to treat (Anderson 2005; Ksiezopolska and Gabaldon 2018; Odds et al. 2003; Perlin et al. 2017; Revie et al. 2018). Antifungal resistance can develop because of regular exposure to increased antifungal micropollutants in the environment nowadays (Stevenson et al. 2022). There are several ways that antifungals can get into the environment. These include, directly applying fungicides to plants as plant protection products and ineffective pharmacological antifungal removal in wastewater treatment systems. Additionally, because of climate change, some regions have higher CO2 levels, longer growing seasons, and warmer temperatures, all of which benefit fungi through quicker evolution and improved growth rates (Chakraborty 2013; Chakraborty and Datta 2003; Roy et al. 2004).

In summary, our study found most of the fungi isolated were resistant to fluconazole and amphotericin B, so they were unlikely to be effective against fungi in the Townsville region. The drugs of choice according to this study would be voriconazole and ketoconazole, as the species of fungi isolated locally displayed susceptibility to these drugs.

Most fungal isolates in this study were identified to species level based on their morphological criteria. Sequencing of the ITS region of the 23sRNA gene was done on representative isolates or those that could not be identified to species level. Both methods only identify to species level. Morphological identification is cheaper but requires a high level of mycological skills as the fungi must be encouraged in a variety of ways to produce fruiting bodies for identification purposes. Sequencing is technically simpler, but more expensive and cannot be carried out in house.

A weakness of our study was the number of fungi isolates tested for MIC was small, so, there might be a chance that these fungi were clonally related. The geographical range used in this study is small, only representing the Townsville region, and additional studies are needed to be able to represent the entire North Queensland. In vitro testing of fungi isolates provides relative but not an absolute indication of clinical response. The concentration of drugs achievable in the cornea might differ according to the pharmacokinetic and pharmacodynamic properties of the medications used. Additionally, host factors (immune responses and genetics) play a role in the in vivo effectiveness of the treatment (Hampson et al. 2019; Pearce et al. 2009). Moreover, the standardized in vitro criteria are not well established in horses for MIC testing, impeding the interpretation of the relationship between the MIC result and clinical outcomes. This method can only forecast the in vivo efficacy of antifungal drugs and the correlation between in vitro testing and successful in vivo treatment of fungi is not well established (Espinel-Ingroff 2003; Odds et al. 1998). Nevertheless, these MIC results can help clinicians when susceptibility tests are not available and as a preliminary guide in choosing the therapeutic protocol for equine keratomycosis and add to the information on fungal flora found in the eyes of horses living in tropical regions of the world.

## Data Availability

Raw Data is available on request.
